# Protective effect of melatonin on alleviating early oxidative stress induced by DOX in mice spermatogenesis and sperm quality maintaining

**DOI:** 10.1186/s12958-022-00977-4

**Published:** 2022-07-18

**Authors:** Teng Zi, YaNan Liu, YuSheng Zhang, ZeLin Wang, ZhiXin Wang, Song Zhan, Zhu Peng, Ning Li, XueXia Liu, FuJun Liu

**Affiliations:** 1 Shandong Stem Cell Engineering Technology Research Center, Affiliated Yantai Yuhuangding Hospital of Qingdao University, Yantai, China; 2 School of Bioscience and Technology, Weifang Medical University, Weifang, China

**Keywords:** Doxorubicin, Melatonin, Spermatogenesis, Sperm quality, Testis

## Abstract

Doxorubicin (DOX) is an effective chemotherapy drug, but its clinical use has adverse effects on male reproduction. However, there are few studies about the specific biological processes related to male reproduction or strategies for improving fertility protection. In this paper, we examined the effects of DOX on spermatogenesis and sperm function, and tested the possible protective role of melatonin (MLT) against DOX’s reproductive toxicity. DOX-treated mice showed signs of significantly impaired spermatogenesis, including vacuolated epithelial cells, decreased testis weights, and lowered sperm counts and motility. DOX also reduced germ cell proliferation (PCNA) and meiosis-related proteins (SYCP3), but this effect could be partially improved with MLT administration. HSPA2 expression was maintained, which indicated that although MLT did not improve sperm motility, it did have a significant protective effect on elongated sperm. IVF results showed that MLT could partially promote two-cell and blastocyte development that was restricted by DOX. MLT reversed DOX-driven changes in the testes, including the antioxidant indices of SOD1, CAT and PRDX6, and the apoptotic indices of BAX and Caspase3. These results suggest that MLT effectively prevents DOX-induced early reproductive toxicity, and increase our understanding of the molecular mechanisms underlying DOX’s effects on male reproduction and the protective mechanism of MLT.

**Supplementary Information:**

The online version contains supplementary material available at 10.1186/s12958-022-00977-4.

## Introduction

Chemotherapy is one of the most effective treatments for cancer patients [1]. However, chemotherapeutic drugs also have many toxic side effects, including on normal cells and tissues [2–4], but the specific phenotypes and mechanisms involved in these changes are not fully understood. Reproductive toxicity is the most common adverse side effect of chemotherapy drugs [5, 6]. With relevance to male reproduction, spermatogenesis (specifically, the development of Leydig cells) can be affected by chemotherapy drugs, which results in abnormal androgen secretion [7]. Spermatogonial and spermatocyte cells are also hypersensitive to chemotherapy agents, with adverse effects on cellular proliferation [8]. These changes can lead to sperm defects, including decreased sperm numbers, reduced sperm motility, and alterations in sperm function, all of which can lead to male infertility [5]. Thus, it is important to understand the basis of and mechanisms underlying the reproductive toxicology induced by different chemotherapy agents and to explore protective mechanisms for patients undergoing chemotherapy.

Doxorubicin (DOX), a widely-used chemotherapeutic agent, can inhibit topoisomerase II activity and induce DNA breaks in cancer cells [7, 9]. In the male reproductive system, DOX-induced testicular toxicity can result in azoospermia [10]. The underlying mechanisms of these changes include chimeric DNA formation and the overproduction of oxygen radicals in testicular cells. DOX can also damage spermatogonial chromosomes, increase germ cell apoptosis, interfere with the sperm maturation process, and induce gene mutations [11–14]. Taken together, these impairments may account for the defect in sperm function and male infertility. DOX has also previously been shown to reduce testicular and epididymal weights in a rat model in a dose-dependent manner as well as to significantly affect rat sperm motility and cause morphological abnormalities [11, 13]. However, limited reports have shown that germ cells were relatively more affected compared to Leydig and Sertoli cells [15, 16]. These changes can mainly be attributed to the increased oxidative stress induced by DOX treatment, which germ cells are most vulnerable to.

Many studies have attempted to protect against DOX-induced testicular toxicity, with mixed results [17, 18]. Melatonin (MLT) is a promising antioxidant agent with a variety of physiological functions, including biological rhythm regulation, gonadal function stability, and maintenance of normal nervous system activities [19, 20]. MLT also has the capacity for immune regulation, as well as antioxidant and antitumor properties [21]. MLT combined with chemotherapy or radiotherapy can not only significantly improve patient survival rates [19], but can also partially offset chemotherapy-induced changes in the male reproductive system [22]. The molecular mechanism of these effects, however, is still not well-understood.

In the present study, we investigated the effects of DOX on spermatogenesis and sperm quality, as well as MLT’s protective effects on male fertility. Spermatogonial cells and spermatocyte cells are more sensitive to DOX than Leydig cells and Sertoli cells, and DOX can significantly affect sperm’s functional capacity. Hence, spermatogenesis and sperm function were selected as the focus of this study in order to examine the specific elements of spermatogenesis and/or subsequent sperm function that were affected by DOX and MLT. We hope our findings will provide important information for further research related to MLT’s protective effects on male fertility during cancer chemotherapy.

## Materials and methods

### Animals

Male ICR mice (6–8 weeks-old, 31–36 g, Beijing Vital River Laboratory Animal Technology Co., Ltd., Beijing, China) were raised in a 12 h light/dark cycle at constant room temperature (RT) (23 ± 1℃) with a regular diet. All animals were bred and handled in accordance with the guidelines for the care and use of laboratory animals in order to reduce the number of animals used and any potential suffering. This study was approved by the Medical Ethics Committee of Yantai Yuhuangding Hospital (No. 2020 − 189).

### Construction of DOX-induced mouse model

The animals were divided into four groups: Group 1 (Control, 105 mice including 35 mice in each treatment group and 5 mice for each time point): Mice were intraperitoneally injected with physiological saline once a week for 5 weeks, and were used as a negative control. Group 2 (DOX treated, 35 mice): Mice were intraperitoneally injected with 10 mg/kg DOX one time. Group 3 (MLT treated, 35 mice): Mice were administered 10 mg/kg MLT intraperitoneally for several consecutive days. Group 4 (MLT + DOX treated, 35 mice): Mice received 10 mg/kg DOX (body weight/day) on day 1 of the experiment and 10 mg/kg of intraperitoneal MLT for several consecutive days. DOX and melatonin dosing were based on previously published articles [23, 24]. Mice were anesthetized for the experiments at different time points (day 3, day 5, week 1, week 2, week 3, week 4 and week 5) following drug administration. All experiments included five mice per group, with one testis fixed for histopathology and the other one fresh-frozen for protein or mRNA extraction. The relative weight of the testis was calculated as a ratio of testis weight/body weight (g/g).

Before euthanasia, all mice had serum collected to measure testosterone concentration quantitatively (using liquid chromatography-tandem mass spectrometry).

Because caudal epididymal sperm are functionally mature, sperm from this region were collected for analysis of parameters and function. Briefly, caudal epididymides were minced in PBS to release sperm into the buffer at 35℃ for 5 min. Sperm parameters were measured using the Computer-aided Semen Analysis System (CASA) (Medealab™, Erlangen, Germany).

Testicular morphology was observed using Hematoxylin and Eosin (H&E) staining. Testes samples were fixed in Bouin’s fluid for 48 h, embedded in paraffin, sliced into sections and de-waxed alternately with xylene and alcohol. After being stained, respectively, in hematoxylin solution and working eosin solution for 1 min, sections were dehydrated and observed under a light microscope (DM LB2, Leica, Nussloch, Germany).

### Western blotting

Testis were ground in liquid nitrogen and extracted with a RIPA lysis buffer (50 mM Tris-HCl pH 7.4 150 mM NaCl, with protease inhibitor cocktail). After determination of concentration, 50 µg proteins from each sample were loaded in 12% gels for sodium dodecyl sulfate-polyacrylamide gel electrophoresis (SDS)-PAGE. Polyvinylidene difluoride (PVDF) membranes were used for gel transference at 100 v for 1 h, blocked with 5% (w/v) skimmed milk for 1 h at room temperature (RT), and incubated with different primary antibodies at 4℃ overnight with gentle agitation (Supplementary Table 1). The membranes were washed with 0.5% (v/v) Tween-20 in Tris-buffered saline (TBS) three times, followed by incubation with horseradish peroxidase (HRP)-conjugated anti-IgG for 1 h at RT. Protein bands were displayed using an enhanced chemiluminescence (ECL) kit (Amersham Life Science, Cleveland, OH, USA). The grey value of each band was analyzed with ImageJ software and indicated the protein expression level. The average grey value of each band was normalized to the ACTB values.

### Immunohistochemistry (IHC)

For immunohistochemistry, sections were subject to dehydration and rehydration with xylene and alcohol, respectively, antigen retrieval with antigen repair buffer in a microwave oven, endogenous peroxidases elimination using 3% (v/v) H_2_O_2_, and non-specific binding with 3% (w/v) bovine serum albumin (BSA) in TBS. Sections were then incubated with the primary antibody overnight at 4℃, washed with TBS three times, and incubated with horseradish peroxidase-conjugated IgG (Zhong-Shan Biotechnology, Beijing, China) at a final dilution of 1:400 for 1 h at 37℃. After 3, 3’-diaminobenzidine (DAB) kit (Zhong-Shan Biotechnology, Beijing, China) treatment and Hematoxylin counterstain, the sections were dehydrated and observed under bright-field microscopy (DM LB2, Leica, Nussloch, Germany). Pre-immune IgG was used as a negative control.

10 fields from each section were selected and the positive reaction sites were captured using an optical microscope and analyzed with image analysis software (Image-Pro Plus 6.0; Media Cybernetics, Silver Spring, MD, USA). The immunostaining images were converted to a grey scale, and a linear combination between the average grey signal intensity and the relative area of positively stained cells was defined as the integrated optical density (IOD).

### Male fertility assay

In vitro fertility (IVF) analysis was performed to assess sperm function. Caudal epididymal sperm were released into 200 µl pre-warmed C-TYH medium for capacitation in a 37℃, 5% CO_2_ incubator for 0.5 h. After counting, about 10^5^ sperm/ml were incubated with oocytes for fertilization in a pre-balanced HTF medium covered with paraffin oil. Normal female mice were super-ovulated with an intraperitoneal injection of 10 IU pregnant mare serum gonadotrophin (PMSG) and human chorionic gonadotrophin (hCG) at 48 h intervals, and oocytes were obtained 14 h after hCG injection. The embryo development rate was defined as a percentage of the two-cell embryos/the number of pronuclei-forming oocytes. The blastocyte rate was defined as a percentage of the blastocytes/ the number of two cell embryos.

### Statistical analysis

All data were displayed as mean ± standard deviation (SD) for triple repeats. All statistical analyses were performed using GraphPad Prism 8 (GraphPad Prism, La Jolla, CA). The mean values were analyzed using one-way analysis of variance (ANOVA). A *p* value less than 0.05 was considered statistically significant.

## Results

### DOX significantly inhibited germ cell proliferation and meiosis

**Fig. 1:**
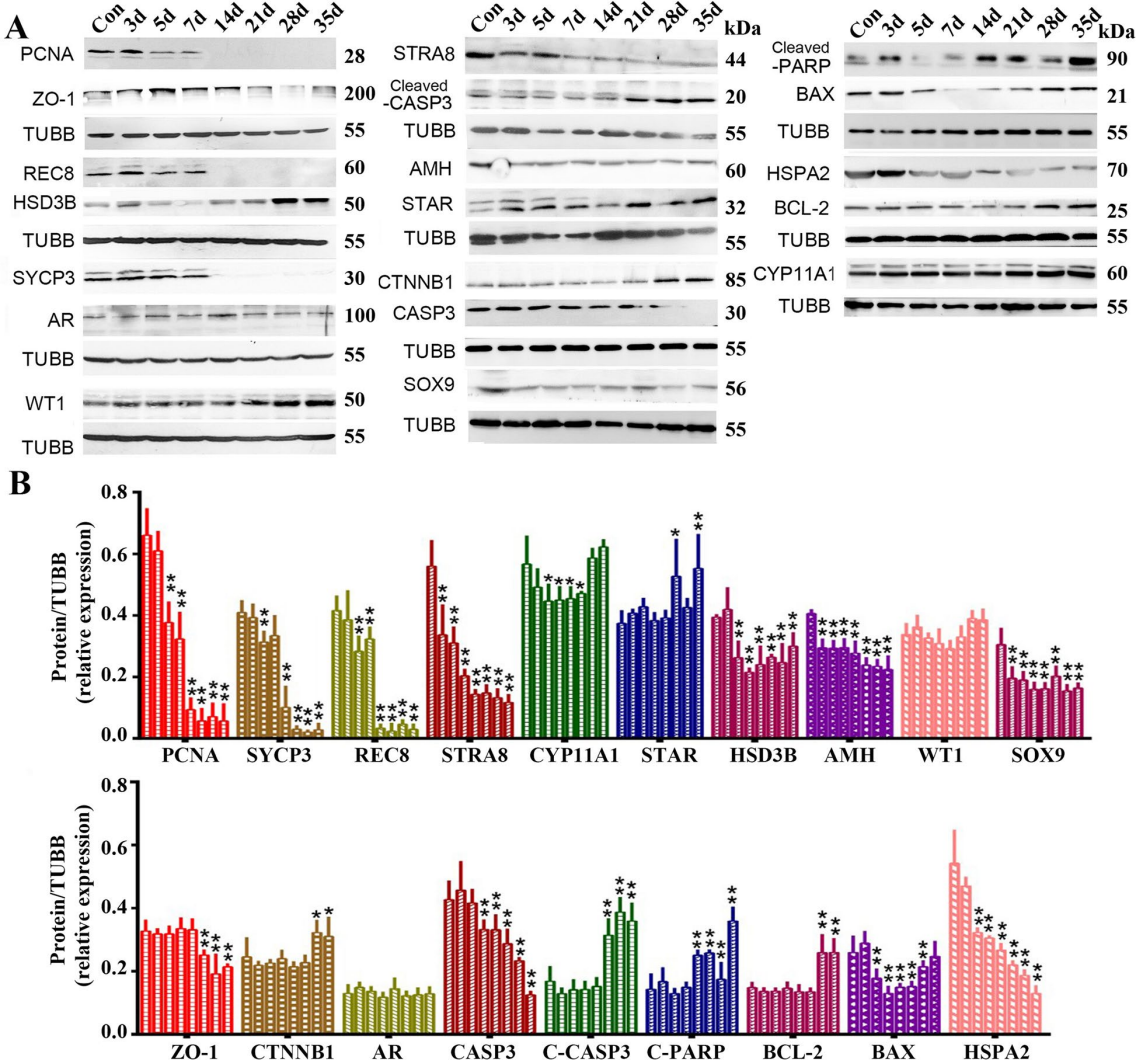
The expressions of key proteins related to spermatogenesis in DOX-treated mice. **A** Representative image of Western blot analysis; **B** Quantitative analysis of detected protein bands, the bars from left to right represented the control, days 3, 5, 7, 14, 21, 28 and 35 after DOX administration. The statistical analyses were performed by One-Way ANOVA, *, *p* < 0.05; **, *p* < 0.01

In order to explore DOX detrimental effects, we checked the expression of key molecules associated with spermatogenesis and sperm quality in group 3 mice testes at different times following DOX administration. As indicated in Fig. 1, PCNA (germ cell proliferation indicator), and SYCP3, STRA8, and REC8 (meiosis-related proteins) were all significantly decreased 14 days after DOX treatment. However, CYP11A1, STAR, HSD3B (steroid secretion-related proteins), ZO-1, beta-catenin (blood-testis barrier-related proteins), and AMH, WT1, and SOX9 (Sertoli cell-related proteins) showed no significant continuous alterations. DOX-treatment significantly lowered the expression of the male fertility-related protein HSPA2. Up-regulated expression of cleaved-caspase 3, cleaved-PARP and Bax indicated increases in apoptotic activity.

### MLT alleviated DOX’s early damage on testicular germ cells

**Fig. 2:**
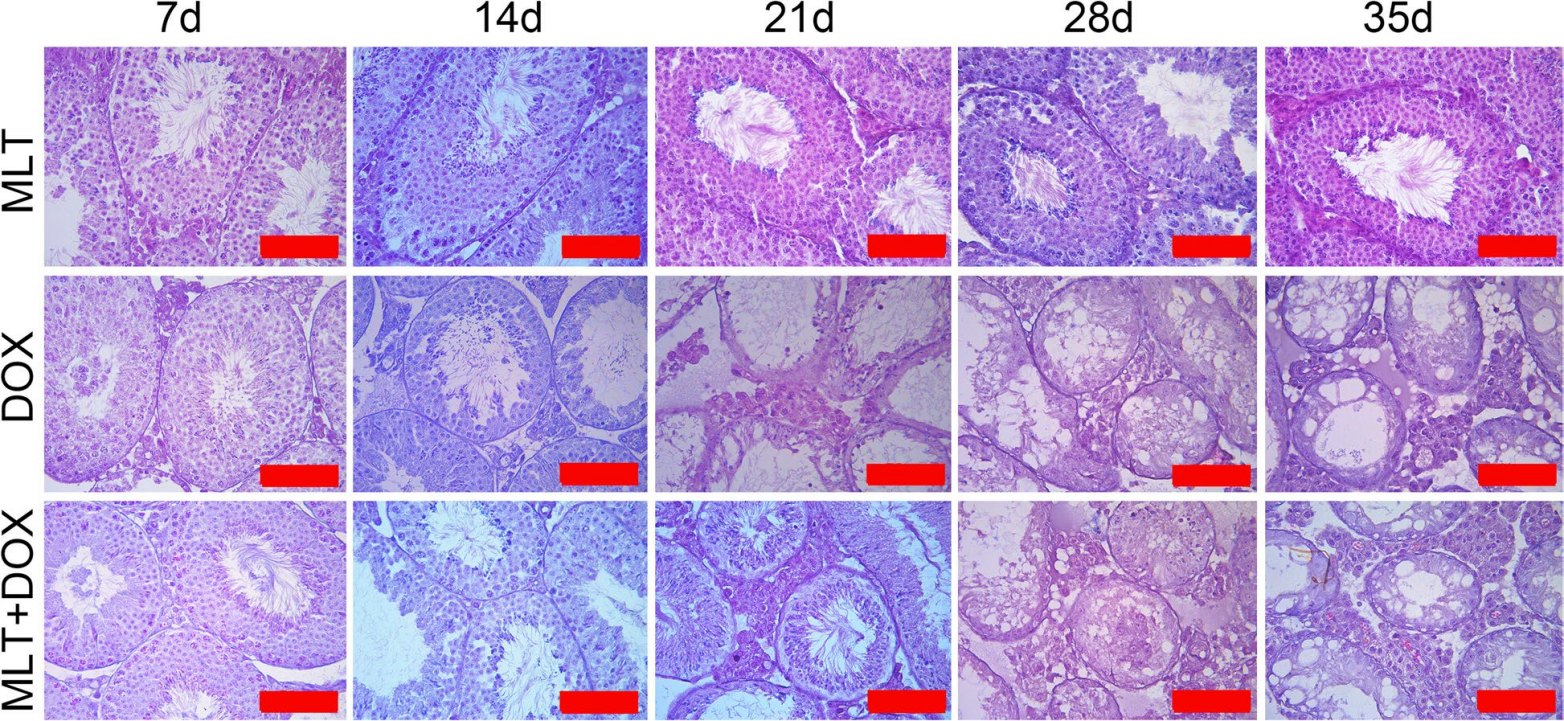
Morphologic analysis of mice testis treated with MLT and DOX at different times. The images were stained by HE. Each bar represented 50 μm

HE staining was performed to analyze testicular morphological changes in the DOX-treated and control groups. The results showed obvious testicular morphology alterations (including thinned germ cell layers, demonstrated vacuolation, and significantly reduced spermatogonia and spermatocyte cells) beginning the 14th day after DOX treatment (Fig. 2). Morphological observations also indicated that germ cells were absent starting 21 days after DOX treatment, which was consistent with decreases in PCNA and SYCP3 expression (Fig. 1). Interestingly, MLT had a protective effect on the early germ cell damage that was induced by DOX. Elongated sperm could be observed on days 14 and 28 following DOX + MLT administration, and a few sperm could also be observed on day 28. However, no apparent sperm were seen on day 35.

**Fig. 3:**
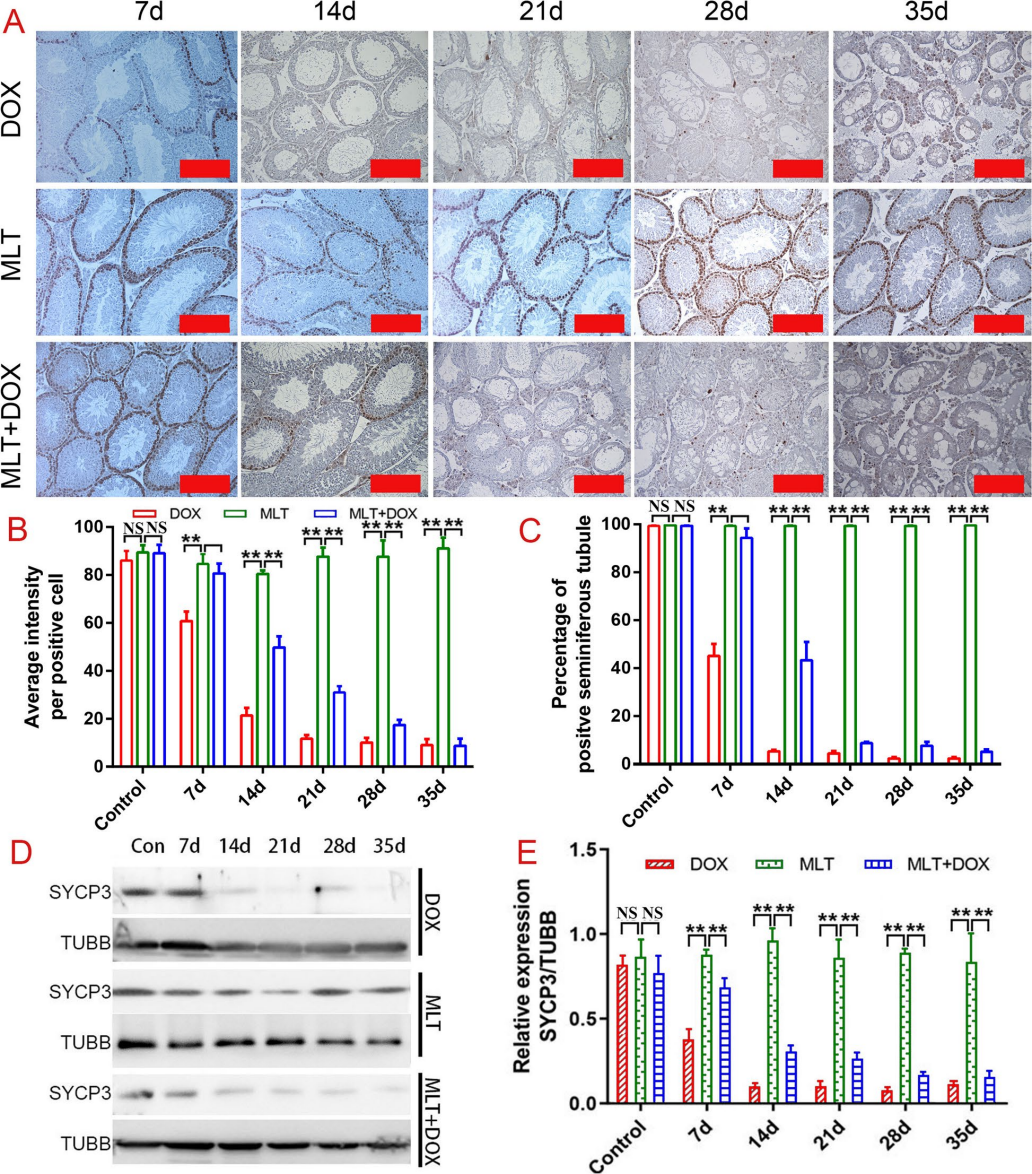
Expressions of PCNA and SYCP3 in mice testes from the different treatment group. Testes from DOX, MLT, and DOX + MLT group at different treatment time were analyzed; **A** immunohistochemistry analysis of PCNA in testes; Average intensity of positive germ cells **B** and percentage of positive seminiferous tubules **C** were counted and analyzed by one-way ANOVA; **D** Western blot analysis of SYCP3 expression in testes; **E** Quantitative analysis of SYCP3 expression relative to TUBB expression by ImageJ software. **, *p* < 0.01; NS: No significance. Each bar represented 50 μm

PCNA was mainly expressed on spermatogonial and spermatocyte cells, and was undetectable 14 days after DOX injection. Although MLT helped maintain PCNA expression until the 14th day after treatment, the decreasing sperm cell number was obvious by day 28. MLT also failed to improve SYCP3 expression on days 28 and 35. These results indicated that MLT could alleviate the earlier DOX-induced changes on testicular germ cells in a sustained manner (Fig. 3).

### MLT significantly protected sperm function rather than sperm motility

**Fig. 4:**
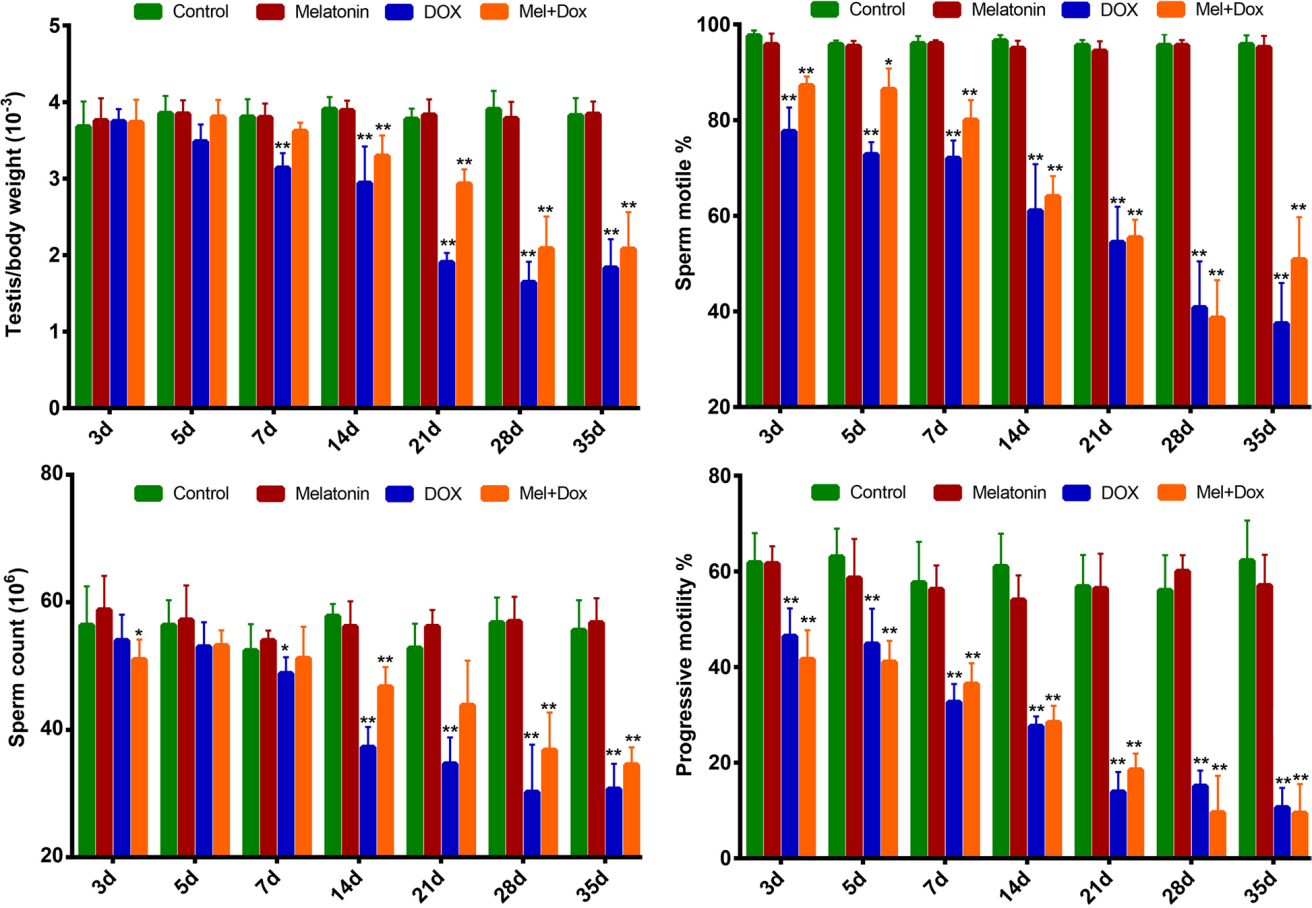
Analysis of testis and sperm quality in control and DOX-treated mice. Ratio of testis/body weight, sperm motile, sperm count and progressive sperm motility were compared among control and DOX-treated mice. Data were compared by one-way ANOVA. *p* < 0.05 was considered significant; *, *p* < 0.05; **, *p* < 0.05

The relative testis weight decreased significantly from day 7 in group 3, while MLT administration slowed weight decline. From the 3rd day onwards, DOX had negative effects on sperm motility rates, sperm counts, and sperm progressive motility. MLT had a certain protective effect on maintaining sperm count, but was not correlated with improvements in sperm motile rates or motility (Fig. 4).

**Fig. 5:**
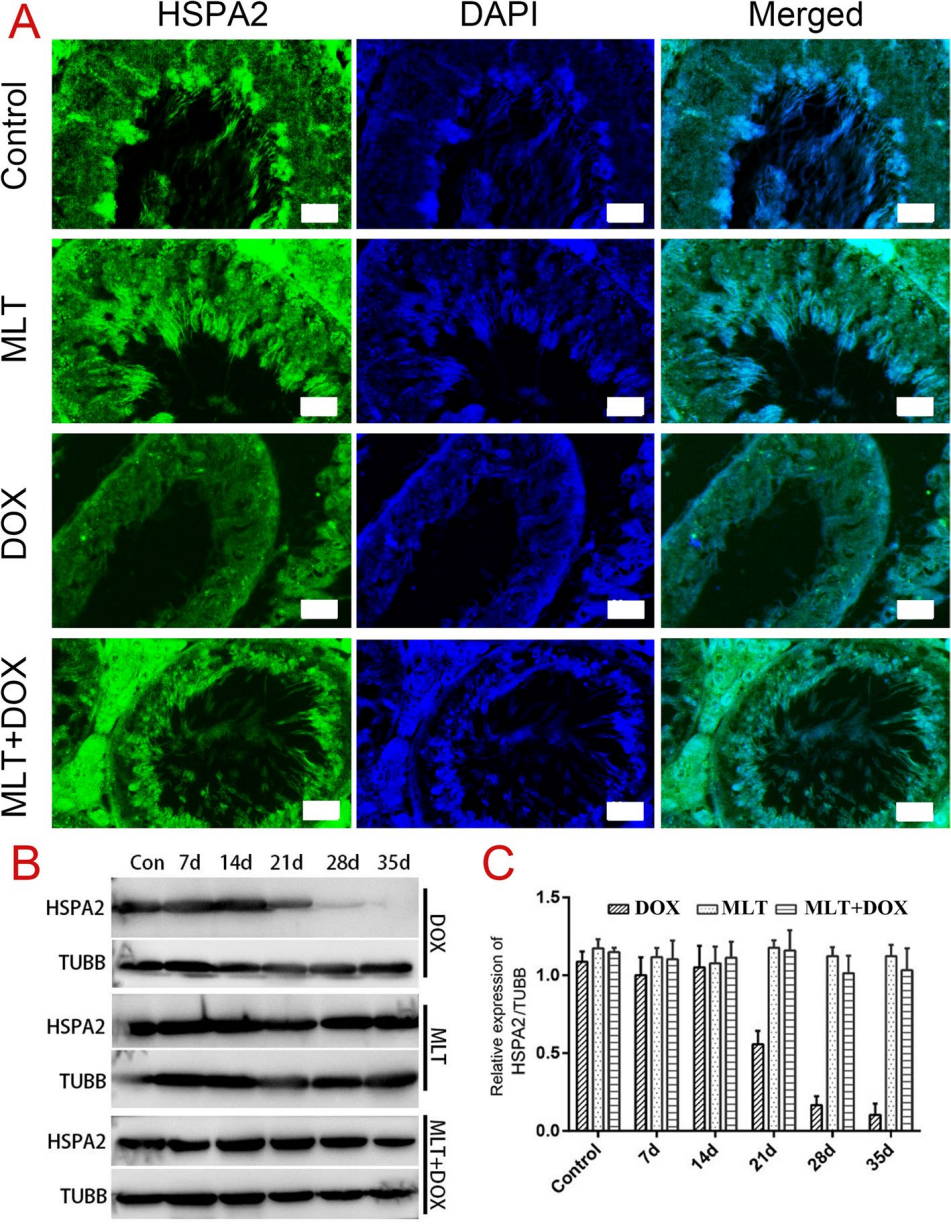
The expression of HSPA2 in DOX, MLT, and DOX + MLT treated mice testis. **A** The cellular expression of HSPA2 in testis after treatment of 14 days; **B** Western blot analysis of HSPA2 at different times of treatment; **C** Quantitative analysis of HSPA2 expression relative to TUBB by ImageJ software. Statistical analysis was performed by one-way ANOVA; **, *p* < 0.01. Each bar represented 20 μm

HSPA2 is an established fertility-related protein that is mainly expressed in spermatocytes and elongated sperm. Compared with group 3, the elongated sperm in the seminiferous tubules in group 4 remained the same until day 14, while the number of other germ cells was significantly reduced. Western blots showed obvious up-regulation in HSPA2 expression after MLT administration, which persisted until the 28th day (Fig. 5). The combined results showed that MLT had a relatively greater protective role on elongated spermatids than on developing germ cells.

**Fig. 6:**
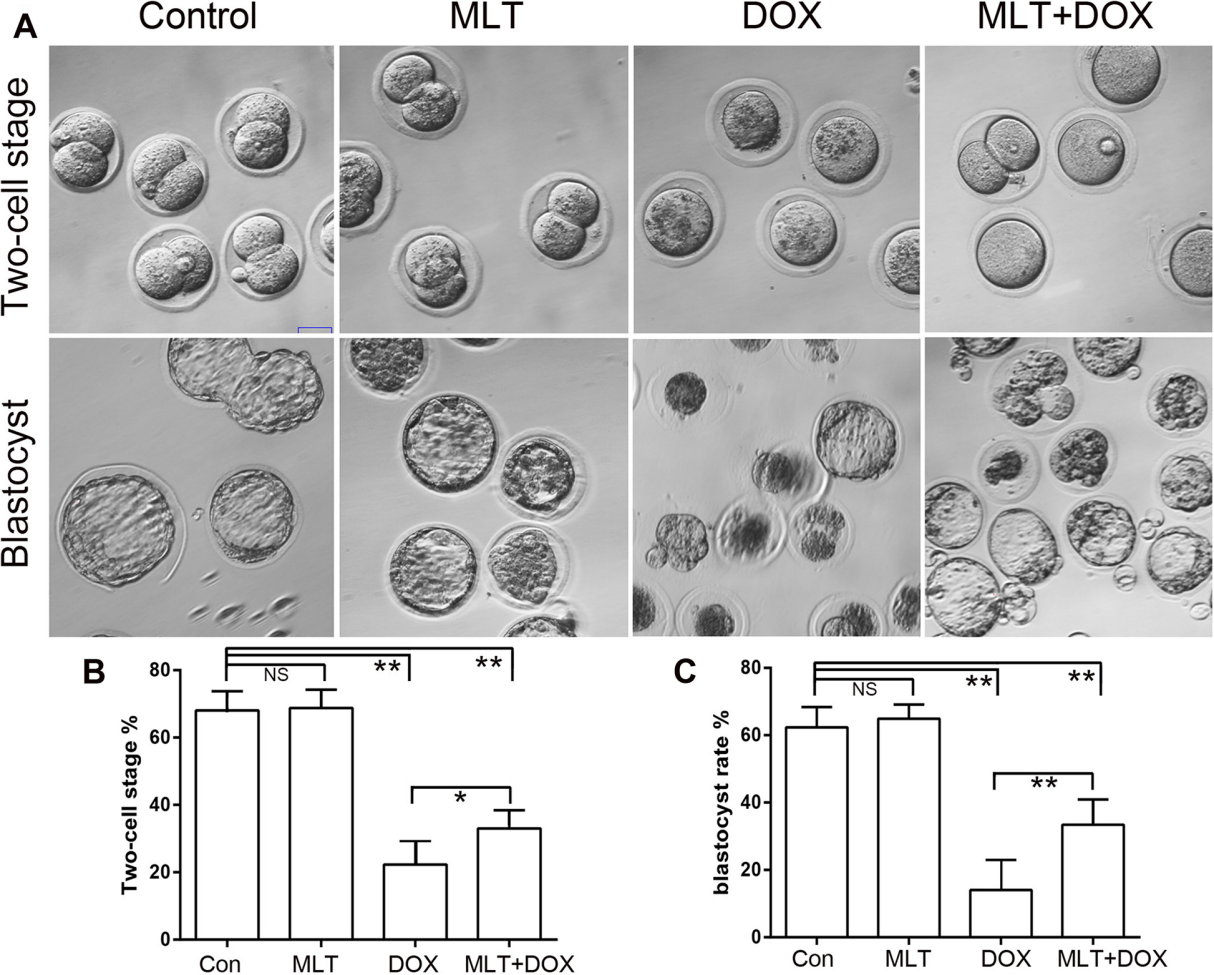
In Vitro embryo development analysis. IVF analysis was performed using epididymal sperm from the control, melatonin, DOX and MLT + DOX group, respectively. Oocytes were collected from normal female mice. DOX, Doxorubicin; MLT, melatonin; All the statistical analyses were performed by ONE-WAY ANOVA;NS. No Significance, **, *p* < 0.001

The IVF experiment using epididymal sperm indicated that the rate of embryo development was significantly reduced in groups 3 and 4. However, MLT administration significantly increased the percentage of two-cell and blastocyte development (Fig. 6).

### MLT could ameliorate apoptosis induced by oxidative stress in DOX-treated mice testis

**Fig. 7:**
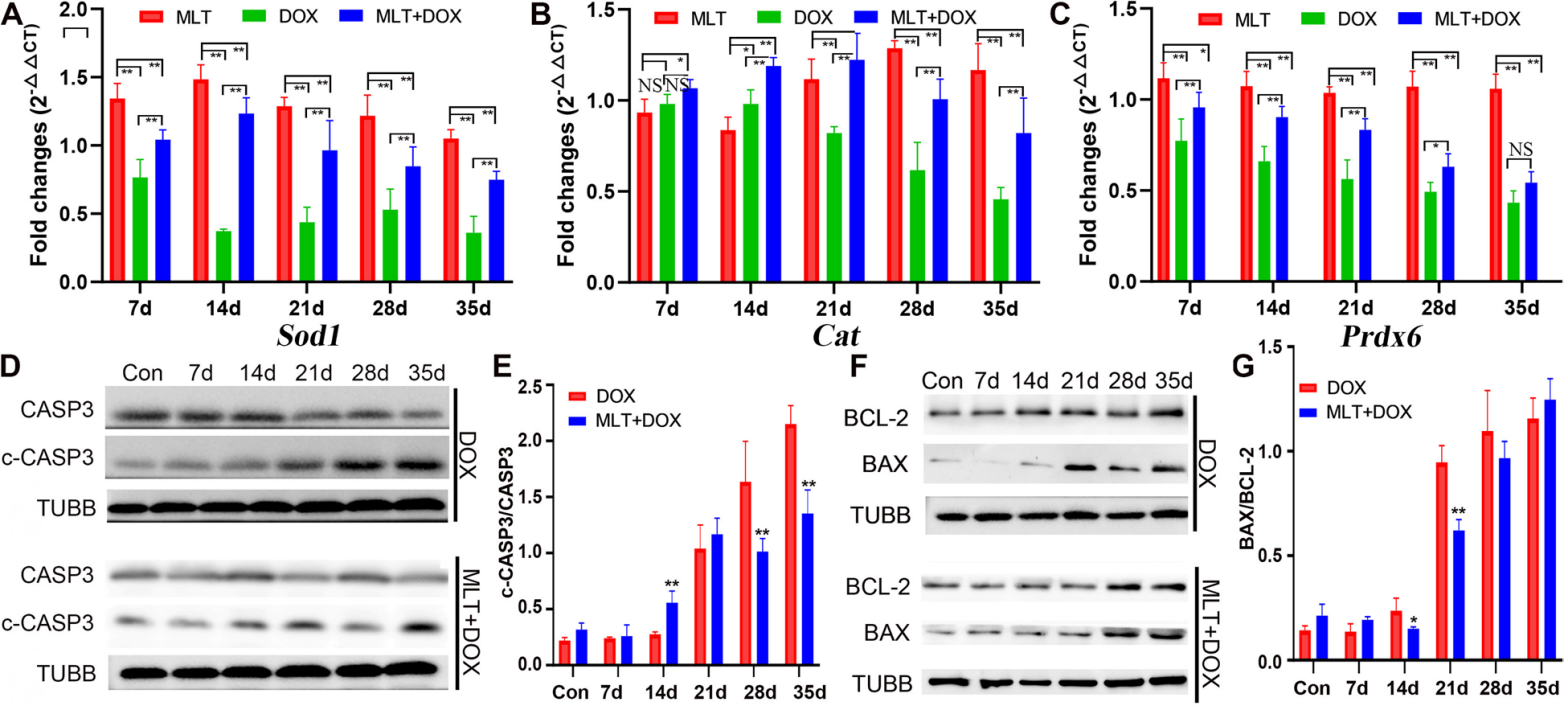
The expressions of antioxidant genes and apoptosis proteins in DOX and MLT + DOX treated mice testes. The expressions of SOD1, CAT and PRDX6 were analyzed by real time RT-PCR and quantified by 2^−△△ct^; The expressions of CASP3, BCL-2 and BAX were analyzed by Western blot and quantified by ImageJ software. DOX, Doxorubicin; MLT, melatonin; All the statistical analyses were performed by ONE-WAY ANOVA; *, *p* < 0.05, **, *p* < 0.001

As shown in Fig. 1, decreased expression of cleaved-Caspase 3 and PARP, and increased expression of BAX beginning on the 14th day after DOX administration suggested the occurrence of apoptosis. In group 4, MLT significantly up-regulated the expression of the antioxidant genes SOD1, CAT and PRDX6 that had been decreased in group 3. Compared with DOX treatment alone, MLT significantly down-regulated cleaved-Caspase 3 expression on days 14, 28, and 35, while the relative decreases in BAX expression were only observed on days 14 and 21 of DOX treatment (Fig. 7).

## Discussion

DOX, a highly effective anti-cancer drug, has toxic effects on reproduction and can cause male infertility [11–15] via multiple mechanisms, including oxidative stress and DNA damage. MLT is thought to have antioxidant effects that can counteract cell damage caused by chemotherapy drugs [19]. In this study, we focused on the spermatogenesis and sperm function in order to investigate the effects of DOX on male reproduction and the protective antioxidant roles that MLT may play in ameliorating DOX-induced changes.

Although DOX has been linked to abnormal testicular function and male infertility [13, 14], neither the specific molecular mechanism of its effects on spermatogenesis nor the possible protective effects of MLT have been well investigated. Based on previous reports, we selected a dose of 10 mg/kg DOX for our study [25]. The body weight of DOX-treated mice decreased dramatically, especially after 14 days post-treatment. The relative testes weights were also significantly reduced beginning the 7th day after treatment, suggesting that DOX may mainly affect spermatogenesis. The testis is an important organ for spermatogenesis, as it is involved in meiosis, steroid hormone secretion and spermiogenesis [26]. The orderly and normal occurrence of these biological events requires normal functioning of Sertoli cells, Leydig cells, and germ cells, which can be characterized by blood-testis barrier formation, steroidogenesis and cell proliferation, respectively. The expression of key molecules related to these biological processes decreased following DOX treatment. Among these molecules, PCNA and SYCP3 showed the most obvious declines in expression from the 14th post-treatment day onward. The results demonstrated that DOX had significant cytotoxic effects on germ cell proliferation and meiosis, which was consistent with the notion that germ cells are more sensitive than Sertoli and Leydig cells to the effects of chemotherapy drugs. This may be because testes have a blood-testis barrier that can protect pachytene spermatocytes, while spermatogonia and the primary spermatocytes are more vulnerable to ROS induced by chemotherapy drugs.

As an important antioxidant molecule, MLT plays a significant role in preventing oxidative damage caused by chemotherapy drugs. In this paper, the protective effects of MLT against DOX, and possible mechanism underlying these effects, were discussed in the context of spermatogenesis and sperm quality. The seminiferous tubules of the testicles showed vacuoles starting 7 days after DOX administration, and MLT treatment could help maintain normal testicular morphology at this time point. The number of germ cells also decreased significantly starting 14 day after treatment, suggesting that DOX had significant effects on spermatogenesis. From day 21 of DOX treatment onwards, testicular morphology changed significantly. Specifically, there were significant decreases in the number of germ cells, but we found that MLT could partially retain elongated spermatids. However, starting 28 days after DOX administration onward, MLT did not improve germ cell loss, suggesting it could only ameliorate early oxidative stress-induced changes [27]. Although MLT treatment maintained SOD1 and PRDX6 expression levels (which showed visible decreases starting 14 days after DOX treatment), their expressions still showed some time-dependent decreases. DOX can affect testicular functioning via oxidative stress and DNA damage [28]. Our results suggested that although ROS might play an important role in the early stage of DOX treatment, DNA damage in germ cells was also a main driver of DOX’s cytotoxic effects on spermatogenesis and these effects may also be related to excessive dosages.

DOX increased the sperm malformation rate while simultaneously decreasing sperm numbers and motility. MLT played a partial role in maintaining sperm numbers but had no obvious protective effects on sperm motile rate or motility. As for the protective effects on spermatogenesis, MLT could improve the decreased expression of PCNA that was induced by DOX on the 7th day and 14th days post-treatment, but it had no obviously ameliorative role after 21 days. Trends in SYCP3 protein expression were consistent with findings from the morphological analysis. DOX is known to induce oxidative stress in the testis. SOD1, PRDX6 and CAT had similar expression trends, suggesting that MLT could rescue early oxidative damage caused by DOX but not DNA damage caused by high doses of DOX (which resulted in damaged spermatogonial cells that can not maintain subsequent spermatogenesis or maturation properly). Taken together, the alterations induced by DOX led to testicular toxicity, which also significantly impeded spermatogenesis.

Interestingly, HSPA2 expression in the testis differed from that of spermatogenesis-related proteins. HSPA2 is an important fertility-related protein and is mainly expressed in elongated testicular spermatids [29]. The expression of HSPA2 remarkably decreased 21 days after DOX treatment, but its expression was significantly improved following MLT administration. The results suggested that MLT could slow DOX-induced oxidative damage on sperm cells. This might account for the rich lipid components in sperm, which are more vulnerable to oxidative stress-related damage [30]. Epididymal sperm were mainly preserved in the caudal epididymis, suggesting that MLT had antioxidant effects on epididymal sperm, and providing important information for further research related to the protective effects of MLT on epididymal sperm maturation. In this study, the fertilization capacity of caudal epididymal sperm was evaluated using IVF. Compared with DOX treatment, MLT significantly improved two-cell development and blastocyst formation rates, indicating a critical protective role of MLT on sperm. Poly (ADP-ribose) polymerases (PARP) is an enzyme involved in various apoptosis-associated cellular processes [31]. DOX treatment led to activation of PARP-1, which induced caspase-independent cell death. BAX expression was also up-regulated in a time-dependent manner. MLT could alleviate the expression of BAX and caspase 3, suggesting the partial protective roles of MLT might work via an anti-apoptosis pathway against BAX and Caspase 3.

## Conclusions

Our experimental results suggested MLT could partially restore testis and sperm function. MTL’s lack of protective effects after 14 days may be due to excessive DOX dosages, leading to excessive depletion of spermatogonial cells. Our findings also suggest that MLT’s protective effects on DOX-induced reproductive changes may mainly work through the alleviation of oxidative stress, and that MLT can’t counter DNA damage. MLT may have a protective role in restoring spermatogenesis and sperm quality in low-dose DOX-treated animal models and is worthy of further study. This paper provides important information for further studies on the reproductive toxicology of chemotherapeutic drugs and the protective effects of MLT.

## Supplementary Information

Additional file 1: **Table S1.** Lists of primary antibodies.

## Data Availability

The datasets during and/or analyzed during the current study available from the corresponding author on reasonable request.

## Abbreviations

ANOVA: One‐way analysis of variance
BSA: Bovine serum albumin
CASA: Computer-aided Semen Analysis system
DAB: 3: 3’-diaminobenzidine
DOX: Doxorubicin
ECL: Enhanced chemiluminescence
hCG: Human chorionic gonadotrophin
HRP: Horseradish peroxidase
IHC: Immunohistochemistry
IOD: Integrated optical density
MLT: Melatonin
PAGE: Polyacrylamide gel electrophoresis
PARP: Poly (ADP-ribose) polymerases
PCNA: Proliferating cell nuclear antigen
PMSG: Pregnant mare serum gonadotrophin
PVDF: Polyvinylidene difluoride
ROS: Reactive oxygen species
RT: Room temperature
SD: Standard deviation
SDS: Sodium dodecyl sulfate
SYCP3: Synaptonemal complex protein 3
TBS: Tris-buffered saline
